# Science and Peace

**Published:** 1871-02

**Authors:** 


### SCIENCE AND PEACE.

These words are perhaps not inseparably associated in the mind—yet the facts which they represent are very closely connected. Peace is essential to the successful prosecution of Science. Amid the turmoil of war there is little time for study, and no knowledge is much valued save that which will aid in the work of destruction. On the other hand, Science, by increasing the amount of wealth and comfort, renders men more anxious to avoid the fatal consequences of war. It is the great humanizer, to which will be owing, if it ever happens, that the sword shall be beaten into the plough share. To Science we owe that its votaries love to cultivate peace.

Science is the child of thought. It is the development into practical results of those great truths which nature has hidden from the unobservant eye, but yields to the efforts of research. It is an appreciative comprehension of the laws which the Creator has imposed upon His creation, and a recognition of the manner in which these laws do or may enure to man's benefit. It is the medium through which God's richest bounties have been bestowed, for it is the parent of art, and the chief, if not the sole means by which mankind has been elevated from a condition of helpless ignorance to the present state of knowledge and civilization.

The man of Science is, then, a High Priest of nature, receiving from above an inspiration, the fruits of which, he should dispense to his fellow-men. He is her interpreter, who, having been admitted to her arcana, is required faithfully to deliver her oracles.

The field of his study is broad and abundantly diversified. From all his realms, whether land or flood, wherever he can find the tracing of her fingers, there he may learn some facts conducive to man's physical comfort or intellectual development. It is his to draw aside the veil, and discover the secret workings of her powerful elements. Unworthy would he be of his office, did he gaze otherwise than with profound gratitude and awe. For the man of Science should, more than others, be imbued with a sincere reverence for and love of Truth. It is with Truth, in one of its highest forms, he has to deal. He must love it, not with a mere languid preference, but with an ardent and intense devotion, which will deem no sacrifice of ease too great to be made in its service. For it, he must forego luxury and leisure, defy persecution, turn a deaf ear to the allurements of pleasure, and spend nights of watchfulness and days of care. The true votary of Science must be no slugard—no dreamer over idle phantasies. W hat is learned to-day must be followed up to-morrow, so that theory may stand attested by an array of well-arrayed facts. Truth must be his watchword and his goal. No system, however beautiful in seeming, should receive his favor, unless it can bear this test. No train of speculation, be it never so ingenious, should entice him from the path marked out by reason, and hedged in by the stern logic of facts. Pursuing this course, he will make progress, though he may not attain a full accomplishment of his purposes. His efforts will not utterly fail, though no great success may strike the senses of the multitude. Blessings will flow from his labors, though he may be denied the privilege of viewing, by prophetic ken, the rich prospects which he opens to his race.

But his love of Truth should not end here. Faithful in its pursuits, he should be equally faithful in delivering it to others. This is not always pleasant or easy to do. Mankind generally are averse to scientific truth. They resist it until it becomes irresistible. Its votaries, who would teach it, they persecute, until from very shame, they can persecute no longer. He who, for instance, devotes himself to the science of Medicine, will have many temptations to swerve from his integrity; for he will soon discover that it is more popular to teach falsehood than truth. He will see the empiric gain, in a few years, more of honor and fame than he can hope to win by a long life-time of toil and study. He will see colossal fortunes amassed by imposing on public credulity, while a meagre competence is all his reward. He will hear theories eulogized which have no foundation in fact, and no support save the flimsiest sophistries, while the truths of science are ridiculed as transcendental phantasms, or the drivellings of dotards. But let him be shaken by none of these things. Let him admit no compromise whatever, even though it should redound to his present advantage. He should feel his mission too noble, his trust too sacred to falter for one moment in the discharge of duty. Let him stand firm to the truth, exhibit it in his character, proclaim it in his teaching, and maintain it earnestly against all opposing persons and creeds.

How much the world stands indebted to men of science, we can never estimate. That we are not savages—that we have in so high a degree, the comforts and blessings of civilized life, we owe to the fact that men have thought, studied, experimented. To them we owe the thousand acts by which labor is saved and convenience provided. To them we owe it that pain is alleviated and life prolonged. Can the men who are thus largely the benefactors of their race, be too much honored or too liberally remunerated ?

